# The Chaperonin-60 Universal Target Is a Barcode for Bacteria That Enables *De Novo* Assembly of Metagenomic Sequence Data

**DOI:** 10.1371/journal.pone.0049755

**Published:** 2012-11-26

**Authors:** Matthew G. Links, Tim J. Dumonceaux, Sean M. Hemmingsen, Janet E. Hill

**Affiliations:** 1 Agriculture and AgriFood Canada, Saskatoon, Saskatchewan, Canada; 2 Department of Veterinary Microbiology, University of Saskatchewan, Saskatoon, Saskatchewan, Canada; 3 National Research Council Canada, Saskatoon, Saskatchewan, Canada; 4 Department of Microbiology and Immunology, University of Saskatchewan, Saskatoon, Saskatchewan, Canada; University of Waterloo, Canada

## Abstract

Barcoding with molecular sequences is widely used to catalogue eukaryotic biodiversity. Studies investigating the community dynamics of microbes have relied heavily on gene-centric metagenomic profiling using two genes (16S rRNA and *cpn*60) to identify and track Bacteria. While there have been criteria formalized for barcoding of eukaryotes, these criteria have not been used to evaluate gene targets for other domains of life. Using the framework of the International Barcode of Life we evaluated DNA barcodes for Bacteria. Candidates from the 16S rRNA gene and the protein coding *cpn*60 gene were evaluated. Within complete bacterial genomes in the public domain representing 983 species from 21 phyla, the largest difference between median pairwise inter- and intra-specific distances (“barcode gap”) was found from *cpn*60. Distribution of sequence diversity along the ∼555 bp *cpn*60 target region was remarkably uniform. The barcode gap of the *cpn*60 universal target facilitated the faithful *de novo* assembly of full-length operational taxonomic units from pyrosequencing data from a synthetic microbial community. Analysis supported the recognition of both 16S rRNA and *cpn*60 as DNA barcodes for Bacteria. The *cpn*60 universal target was found to have a much larger barcode gap than 16S rRNA suggesting *cpn*60 as a preferred barcode for Bacteria. A large barcode gap for *cpn*60 provided a robust target for species-level characterization of data. The assembly of consensus sequences for barcodes was shown to be a reliable method for the identification and tracking of novel microbes in metagenomic studies.

## Introduction

Molecular barcoding is a strategy for cataloging biodiversity through identification and differentiation of organisms using DNA sequencing. Barcodes are relatively short, specifically defined DNA sequences used to identify organisms by comparing the barcode sequence from an unknown sample to a collection of sequences from known reference samples. In order to facilitate molecular barcoding across life the International Barcode of Life (iBOL) project has developed a framework for evaluating potential barcodes [1].

Criteria for barcodes include that they must be universal in the taxa of interest, allowing the development of broad-range (“universal”) PCR assays. The existence of reference sequence data from vouchered samples derived from curated specimen collections is required in order to permit robust identification of sequences. For discrimination of taxa it is also essential that inter-specific sequence distance for the barcode sequence be greater than intra-specific distance. This separation between the average intra-specific and inter-specific distance for a given locus defines the “barcode gap” [2]. Gap size is a critical characteristic for any proposed barcode since it is the key determinant of confident resolution of taxa. For example, Schoch *et al.* [3] recently demonstrated that the ITS region offers a superior barcode target for fungi compare to the commonly used 18S rRNA target since it features a larger barcode gap, making species discrimination more robust.

For prokaryotes, it follows that a barcode locus that meets the iBOL criteria, and is suitable for cataloging biodiversity through the examination of individual specimens, would be a powerful tool for barcoding in communities of microorganisms. Confident resolution of taxa is paramount in either application. The gene encoding the small subunit (16S) ribosomal RNA has been used extensively in gene-centric metagenomic studies of microbial communities. Despite positive features that have led to its status as the *de facto* barcode for Bacteria (universality, many sets of broad-range PCR primers targeting different variable regions, large reference database), the 16S rRNA sequence often fails to provide sufficient information for species-level identification [4], and the occurrence of multiple, identical or nearly identical copies per genome complicates its use as a target for quantification. 16S rRNA sequence based metagenomic studies are commonly limited to reporting of taxa at the genus level or above [5].

Protein coding genes have long been recognized as providing superior resolution of closely related bacterial taxa compared to 16S rRNA [4], [6], [7], and despite statements to the contrary [8], one of these protein coding genes has been demonstrated to provide an alternative “universal target” for bacteria. The gene encoding the 60 kDa chaperonin protein (*cpn*60) found in Bacteria and Eukaryotes has been established as a target for the detection, identification and quantification of microorganisms [9]–[16], as well as for gene-centric metagenomic profiling of microbial communities [17]–[26]. An set of broad-range PCR primers that amplify a region of the gene (the Universal Target, UT) that is generally 552–558 bp [14], [27], and cpnDB, a curated sequence database [28], enhance its utility and contribute to its status as a potentially preferred barcode for Bacteria.

We performed a barcode analysis of the *cpn*60 UT and several regions of the 16S rRNA gene that are widely exploited in systematics and microbial ecology. We show that the barcode gap for the *cpn*60 UT is largest of those examined, and that combined with the length of the target region, facilitate the use of a *de novo* assembly strategy for the formation of operational taxonomic units (OTU) that include the entire length of the target sequence. Finally, we present an approach for the evaluation and optimization of metagenomic assemblies, and demonstrate its application in an examination of the results of assembly of a synthetic community of cloned *cpn*60 UT sequences. The results of this work are discussed in terms of their implications for overcoming some of the limitations of 16S rRNA based sequencing for high resolution profiling of microbial communities, and the characterization of communities where novel taxa are likely to be encountered.

## Materials and Methods

### 16S rRNA and *cpn*60 Sequences

A list of completed BioProjects for bacterial genomes [29] was obtained from the NCBI GenBank RefSeq FTP site (circa 12 April 2012). Each GenBank file was processed to extract DNA sequence data as FASTA, Taxon ID, and gene annotations. The resulting GFF files were parsed in order to identify 16S rRNA and *cpn*60 genes, and the DNA sequences of the genes were extracted. Taxon IDs were used to look up the species name and lineage using the NCBI Taxonomy database. Identification of gene annotations for 16S rRNA and *cpn*60 were based on a list of possible annotations ranging from InterPro annotation to explicit keyword sequences. In cases where multiple gene copies were annotated within a single genome all copies were extracted and used in the subsequent analyses.

### Definition of Putative Bacterial Barcode Regions

Predicted annealing sites of PCR primer pairs for amplification of commonly targeted variable regions were used to delineate putative barcode regions within the 16S rRNA gene. Primers used to amplify V1–V3 and V3–V5 were from the Human Microbiome Project (16S 454 Sequencing Protocol version 4.2.2, http://www.hmpdacc.org/doc/16S_Sequencing_SOP_4.2.2.pdf). Two additional regions (V2–V4, and variable region V6) were delineated using established primers [5]. An alternate, shorter version of the V6 region was identified using primers from Hummelen *et al.* [30]. For *cpn*60, the universal target (UT) region corresponding to nucleotides 274–828 of the *E. coli* 60 kDa chaperonin gene was used for barcode gap analysis [27]. Primer sequences and corresponding gene regions are shown in Table 1.

**Table 1:** Definition of barcode regions based on established PCR primers.

Gene	Target region	*E. coli* nucleotides	Primer sequence (5′–3′)	Reference
16S rRNA	V1–V3	27–534	27F AGAGTTTGATCCTGGCTCAG534R ATTACCGCGGCTGCTGG	HMP 16S 454Sequencing Protocol version 4.2.2
	V2–V4	101–806	AGYGGCGIACGGGTGAGTAAGGACTACARGGTATCTAAT	[5]
	V3–V5	357–926	357F CCTACGGGAGGCAGCAG926R CCGTCAATTCMTTTRAGT	HMP 16S 454Sequencing Protocol version 4.2.2
	V6	907–1073	AAACTCAAAKGAATTGACGGACGAGCTGACGACARCCATG	[5]
	V6-alternate	985–1078	L-V6 CAACGCGARGAACCTTACCR-V6 ACAACACGAGCTGACGAC	[30]
*cpn*60	UT	274–828	H279 GAIIIIGCIGGIGAYGGIACIACIACH280 YKIYKITCICCRAAICCIGGIGCYTT	[14]

### Extraction of Barcode Sequences from Whole Genome Sequences

Full length sequences for 16S rRNA and *cpn*60 were aligned with the RDP aligner [31] or ClustalW [32], respectively. Primer annealing sites were identified manually in each alignment using the eBioX alignment viewer (http://www.ebioinformatics.org/ebiox/) and the predicted amplicon sequence between the annealing sites was extracted. Extracted sequences were processed to remove gaps and then subjected to a second round of multiple sequence alignment by the same algorithm to ensure the best alignment of the putative barcode region for distance calculations. Multiple sequence alignments were converted to PHYLIP format and analyzed with DNADIST to calculate pairwise distances (F84) between all sequence pairs [33]. DNADIST output was parsed to partition intra-specific and inter-specific distances, and histograms were plotted using Excel.

### Synthetic Community Sequencing

A previously described mixture of 20 cloned *cpn*60 UT sequences of human vaginal bacteria with pairwise nucleotide sequence identities of 56–96% was used as a synthetic microbial community for sequence assembly experiments [34]. An equimolar mixture of the 20 plasmids was subjected to *cpn*60 universal primer PCR and pyrosequencing on the Roche GS-FLX Titanium platform. Preparation of amplicon libraries for sequencing was done using established protocols [35].

## Results

### Identification of Vouchered 16S rRNA and *cpn*60 Sequences

Using all RefSeq bacterial genomes in GenBank as a starting point, 1,394 bacterial genomes were identified where both 16S rRNA and *cpn*60 genes could be identified based on annotation. BioProject descriptions provided voucher information for the strain sequenced. A total of 983 species, including at least one representative from each of 21 phyla were included, with the majority (92%) of the genomes belonging to Proteobacteria (48%), Firmicutes (21%), Actinobacteria (12%), Bacteroidetes (5%), Cyanobacteria (3%), or Spirochaetes (3%). Nine records had Taxon IDs corresponding to “unknown” phylum in the NCBI taxonomy (Table S1). Numbers of annotated 16S rRNA genes per genome ranged from 1 to 15 (median = 3), and the number of *cpn*60 genes per genome ranged from 1 to 7 (median = 1). All annotated paralogs of *cpn*60 and 16S rRNA genes were identified and used in distance calculations.

Extraction of the 16S rRNA records from the GenBank annotation was relatively straightforward based on matching of rRNA genes with “16S” in their annotated names. By contrast there was no single annotation characteristic sufficient to recognize the *cpn*60 genes. Thus it was necessary to use keyword matching on gene names (“cpn60”, “groEL”, “hsp60”, “60 kDa chaperonin”, “chaperonin 60”, etc.) in order to extract *cpn*60 sequences.

### Barcode Gap Analysis

**Figure 1:**
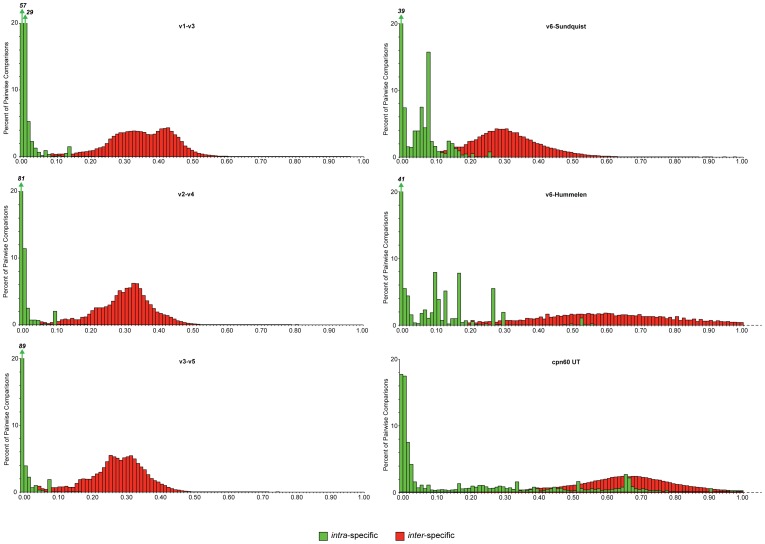
Barcode gaps for candidate targets. Barcode gap analysis of potential barcodes derived from the 16S rRNA and *cpn*60 genes. Each panel shows the distribution of inter- (red) and intra-specific (green) distances in terms of percent of the total number of comparisons made (see Table 2). In cases where percent values exceed 20, the actual value is indicated above an arrow on the relevant bar in the chart. For both V6-alternate and *cpn*60 UT, only distances up to 1.00 are plotted.

The barcode gap analysis of all 16S rRNA regions and the *cpn*60 UT is summarized in Figure 1 and Table 2. The data from 1,394 complete RefSeq bacterial genomes allowed for thousands of intraspecific comparisons for each target and nearly 2 million and 16 million interspecific comparisons for *cpn*60 and 16S rRNA, respectively. Data shown is from distance calculation using the F84 method. Other methods for calculating distance (Kimura’s 2-parameter model, and the Jukes and Cantor model) yielded similar observations both between and within barcodes, and did not affect the conclusions (data not shown). Barcode gaps for 16S rRNA ranged from 0.26 (V6) to 0.35 (V1–V3), with the exception of the ∼75 bp V6-alternate region [30], which had a gap of 0.59. The *cpn*60 UT gap was 0.61 (Table 2). The intra-specific distance distributions for 16S rRNA V1–V3, V2–V4 and V3–V5 were the most narrow, with more than 50% of pairwise comparisons in the range of 0.00 to 0.01 (Figure 1). This was particularly true for V2–V4 and V3–V5 where >80% of the intra-specific comparisons were 0.00–0.01. The intra-specific distance distributions for both V6 targets, and the *cpn*60 UT were relatively enriched in their right-hand tails. The *cpn*60 UT had the highest median intra-specific (0.07) and inter-specific (0.68) distances (Table 2).

**Table 2:** Barcode gap analysis for 16S rRNA and *cpn*60 targets.

				Intraspecific				Interspecific			
Genetarget	Region	Average length (bp)^1^	Barcode gap^2^	# comparisons	Min. distance^3^	Max. distance^3^	Median distance^3^	# comparisons (Millions)	Min.distance^3^	Max.distance^3^	Median distance^3^
16S rRNA	V1–V3	490	0.35	81247	0	0.30	0.00	15.8	0	0.97	0.35
	V2–V4	666	0.31	81247	0	0.22	0.00	15.8	0	0.83	0.31
	V3–V5	551	0.28	81247	0	0.17	0.00	15.8	0	0.80	0.28
	V6	127	0.26	81247	0	0.31	0.04	15.8	0	2.88	0.30
	V6-alternate	75	0.59	81241	0	0.78	0.02	15.8	0	5.91	0.61
											
*cpn*60	UT	556	0.61	3803	0	5.57	0.07	1.7	0	5.89	0.68

1 Median length of the target region, between amplification primer annealing sites.

2 Barcode gap is the difference between the median inter-specific distance and median intra-specific distance.

3 Distance is expressed in terms of substitutions/site.

**Figure 2:**
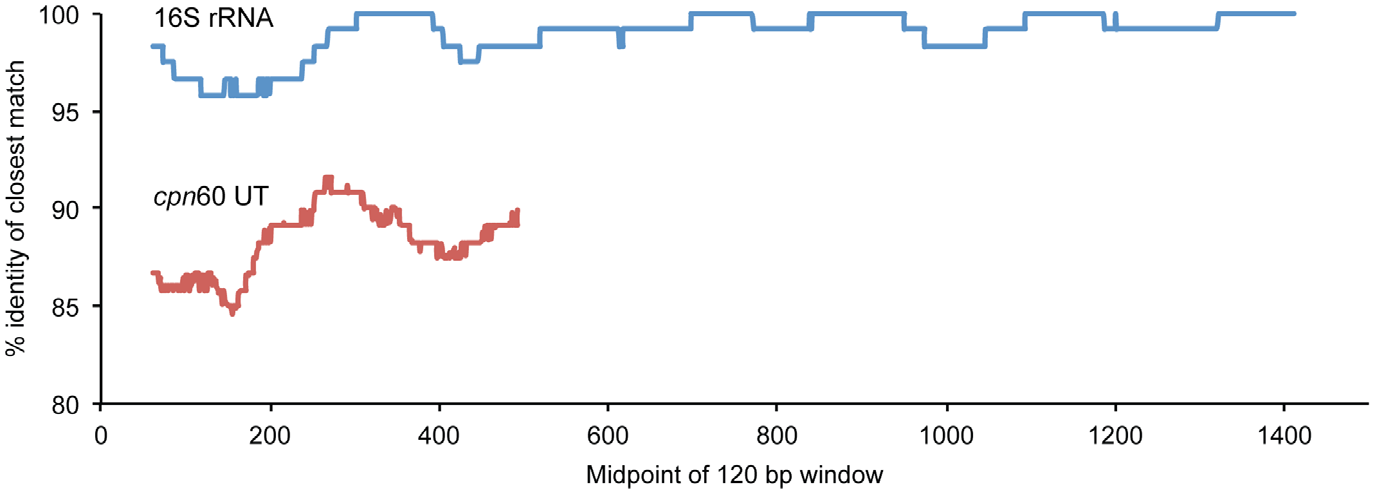
Sequence diversity across the 16S rRNA gene and *cpn*60 UT. Median percent identity of each of sequence to its nearest neighbour among the 16S rRNA *cpn*60 UT sequences from 1,394 bacterial genomes. Median percent identity was calculated for each 120 bp window along the length of the targets and identity values are plotted for the midpoint of each window. Due to target length variation, particularly among 16S rRNA genes, data is shown for windows for which at least 95% of the genomes could be included.

The distributions of inter-specific diversity in the *cpn*60 UT and 16S rRNA gene were determined by calculating the percent identity of each sequence to the next closest sequence. Figure 2 shows the average (median) percent identity between sequences in all 120 bp windows across the targets. Most of the *cpn*60 sequences (87%) were 552–558 bp in length. Diversity along the target length was also remarkably uniform, with median percent identities ranging from 82 to 92%, with most (82%) below 90% identity. Variable and conserved regions of the 16S rRNA gene were visible in the distribution of diversity across the full length of the gene (Figure 2) with conserved regions appearing as stretches of windows with a median identity at or near 100% between sequences. The lowest median inter-sequence identities for the 16S rRNA gene were approximately 96%, and were observed near the 5′ end of the gene, corresponding to variable regions V1 and V2.

### Assembly of *cpn*60 UT Amplicon Sequences

Based on the large barcode gap, target length and uniformity of sequence diversity within the *cpn*60 barcode, optimization of *de novo* assembly of OTU from 454 pyrosequencing data was investigated. A synthetic community comprising 20 cloned *cpn*60 universal targets ranging from 56% - 96% pairwise identity was subjected to universal primer amplification and sequencing with the 454 Titanium pyrosequencing platform. Sequence data was obtained from both ends of the amplicon. A total of 3,437 reads were obtained for the synthetic community with a median read length of 394 (NCBI Sequence Read Archive accession SRR531430). The resulting Standard Flowgram Format (SFF) file was used as input for the gsAssembler in cDNA mode (v2.3, 454 Life Sciences, Branford CT) to form an initial set of OTU. For sequence assembly we focused on the effects of two key parameters: minimum overlap length (-ml) and minimum overlap identity (−mi). Assemblies of the data were conducted, using combinations of minimum overlap length settings of 50, 100, 150, 200, 250, 300, 350 and 400 nucleotides and minimum overlap identity values from 90–99%.

For each assembled OTU, the consensus sequence was evaluated in terms of the extent to which it represented all of the component sequences that were assembled into the OTU. The results of this evaluation were expressed in the form of sensitivity and specificity metrics as follows. Each component sequence and the consensus sequence were compared using wateredBLAST [18] to the *cpn*60 UT sequences of the clones that comprised the synthetic community. “True positives” were individual sequences from the OTU that matched the same reference sequence as the consensus sequence assembled for the OTU, whereas “false positives” were those sequences that were incorrectly placed in the OTU being evaluated (i.e. they matched a different reference sequence than the OTU consensus). “True negatives” were defined as those component sequences that were correctly placed into OTUs other than the one being evaluated, and “false negatives” were identified as sequences that matched the same reference as the consensus for the OTU but had been assembled into other OTUs. Thus for each OTU the specificity was calculated using Equation 1 and the sensitivity for each OTU using Equation 2.

Equation 1. Specificity of an OTU consensus sequence.
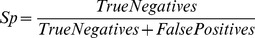



Equation 2. Sensitivity of an OTU consensus sequence.
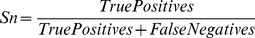



By definition, both sensitivity and specificity of an OTU are values between 0 and 1, with a perfectly assembled OTU having Sp = 1 and Sn = 1 (i.e. no false positives or false negatives). By representing the accuracy of each OTU as a point in a 2-dimensional plane where one axis represented specificity and the other axis sensitivity it was possible to describe the error for a single OTU in terms of Euclidean distance from its coordinates to the optimal coordinates of (1,1) (Equation 3). The total error for an assembly was then calculated by summing the error associated with each OTU in the assembly (Equation 4).

Equation 3. Residual error associated with an OTU consensus sequence.
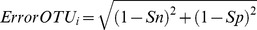



Equation 4. Total error of an assembly.
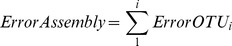



**Figure 3:**
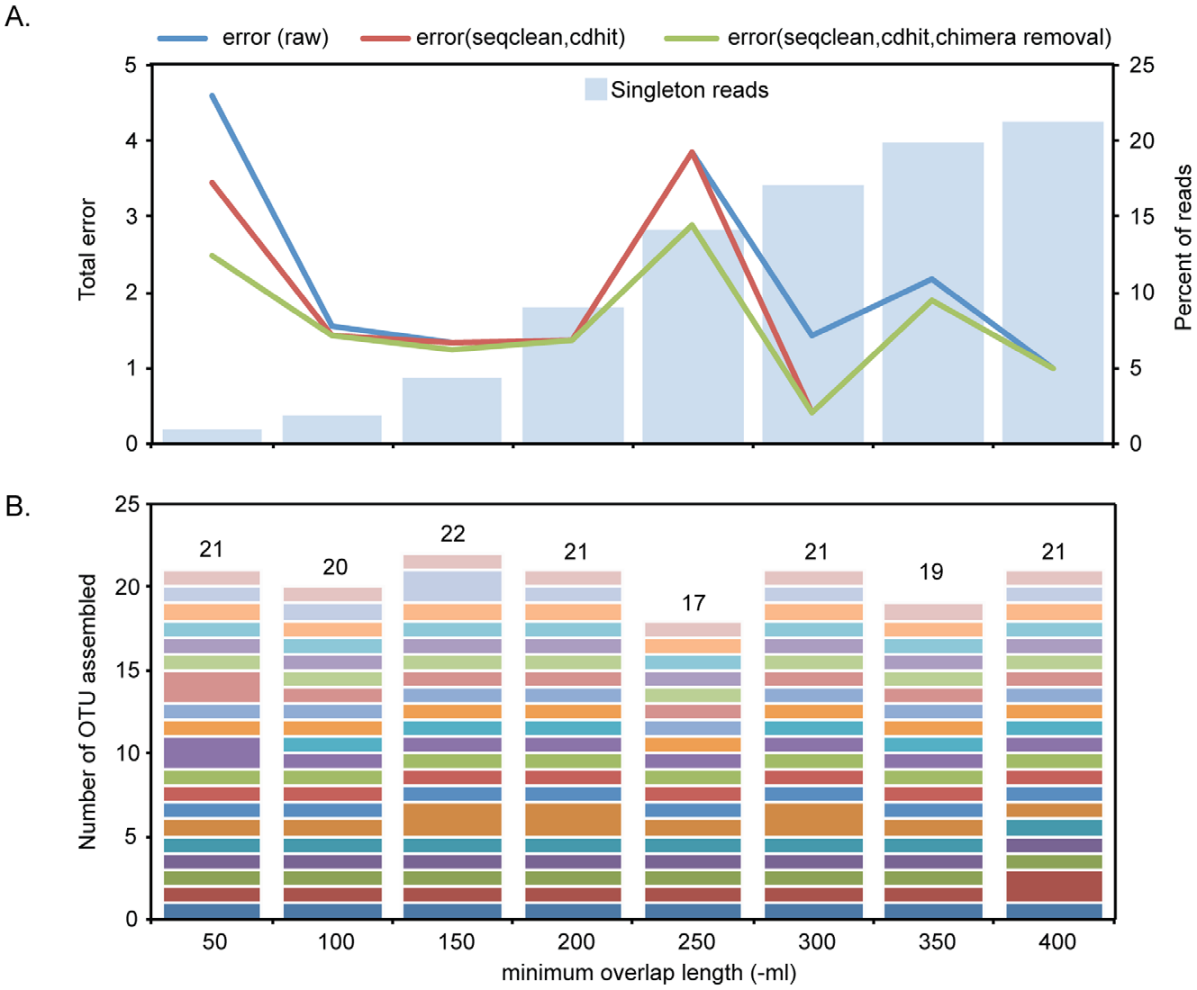
Error trade-offs in OTU assembly optimization. A. Total error (left ordinate) for *de novo* assemblies of *cpn*60 UT sequence reads from a synthetic community of 20 cloned targets, using a minimum identity value of 92% and a range of minimum overlap lengths (50–400 nucleotides). Raw total error (blue line), as well as error remaining after post-assembly primer trimming and clustering (red line), and after chimera removal (green line). Light blue bars indicate the percent of sequence reads identified as singletons in each assembly (right ordinate). B. Number of OTU assembled at each minimum overlap length. Each coloured segment of the stacked bar indicates a different member of the panel of 20 community members. The total number of OTU assembled is indicated on the top of each stack.

In general, minimum identity values of >94% resulted in over-splitting of OTU, regardless of the minimum length parameter. For example, setting the minimum identity parameter at 98% resulted in the assembly of 21 to 25 OTU across the range of minimum overlap settings. For minimum identity values ≤94%, the total error for each assembly varied with the minimum length parameter. Figure 3 shows the results of assemblies of the synthetic community data over a range of minimum overlap lengths with minimum identity 92%. The number of reads identified by the gsAssembler as singletons increased consistently with increasing minimum overlap length, to a maximum of 21% of the reads at -ml = 400, a value that exceeded the median read length of 394 (Figure 3A).

The impact of post-assembly trimming of amplification primer sequences was also investigated. Following assembly, the universal PCR primer sequences were removed using SeqClean (http://compbio.dfci.harvard.edu/tgi/software/) and the sequence data was clustered at 100% identity using CD-hit [36] to combine OTU that were identical once primer sequences were removed. Chimeric OTU were identified as those where the 5′–150 bp and 3′–150 bp matched different sequences in the reference data set. This post-assembly clean up never increased total error, and routinely reduced it, although the amount of error reduction accounted for by primer trimming, clustering and chimera removal was less than that resulting from the optimization of assembly parameters (Figure 3A).

A consistent, small amount of total error was observed across minimum overlap lengths of 100, 150 and 200 (1.44, 1.24 and 1.37 respectively), with almost no reduction in error following post-assembly clean up. The lowest error value observed was for -ml = 300 (0.41 total error), although in this assembly, 17% of the reads were lost as singletons. The total numbers of OTU resulting from the assemblies is shown in Figure 3B.

Assembly of only the 20 expected OTU sequences was achieved with -ml = 100. This included the correct assembly of OTU corresponding to templates *Lactobacillus gasseri* and *Lactobacillus johnsonii* N2, which are 96% identical over their *cpn*60 UT sequences. Eighteen of the 20 OTU sequences included the full-length *cpn*60 UT. OTU corresponding to synthetic community members *Lactobacillus johnsonii* N2 and *Streptococcus* sp. N1 were incomplete (305 and 214 bp respectively). Most of the error observed in the assemblies shown in Figure 3 was due to low sensitivity (i.e. high false negatives). All assembled OTU consensus sequences were 100% identical to the corresponding input *cpn*60 UT template sequences.

With a minimum overlap length of 150, a similarly low total error was observed, but 22 OTU were generated including 2 OTU corresponding to each of *L. gasseri* and *S. gallolyticus*. A comparison of these OTU pairs showed that in both cases there was a full length OTU 100% identical to the reference sequence formed, however in both cases the full length OTU still had a *cpn*60 UT primer sequence on its 5′ end. The shorter sequences were each variant in a single position. In the case of *S. gallolyticus* the shorter sequence had an incorrect single nucleotide deletion and in for *L. gasseri* the last nucleotide of the shorter sequence was incorrectly a T.

## Discussion

*cpn*60 (also known by synonyms GroEL and Hsp60) is a molecular chaperone conserved in Bacteria and in Eukaryotes [37]. The *cpn*60 UT sequence, accessible by PCR using degenerate broad-range (“universal”) primers has been shown to provide resolution of closely related taxa at the species and subspecies level [9]–[11], [14], [38]–[41]. The utility of the *cpn*60 UT sequence as a robust tool for detection, identification and quantification of microorganisms is well established, and it has already been implemented in the development of diagnostic tools based on a variety of technologies including quantitative PCR [16], [42], hybridization on solid substrates [11], [13], [43], and suspension arrays [34]. The success of *cpn*60-based diagnostics is a direct result of the sequence diversity of the barcode, and its length, which provides an abundance of informative sequence differences evenly distributed along the length of the target. Even closely related taxa have sufficient sequence differences to allow their discrimination with confidence. Recently, Verbeke *et al.* [6] demonstrated that unlike 16S rRNA sequences, *cpn*60 UT sequence identities alone are strong predictors of whole genome sequence relationships.

The demonstrated utility of the *cpn*60 UT led us to investigate whether it could be evaluated as a DNA barcode using the iBOL framework. In order to fulfill the requirement for vouchered reference data, we limited our analysis to bacteria for which complete sequences were available in NCBI BioProjects. The first significant difference between 16S rRNA and *cpn*60 targets was encountered at the sequence alignment stage. Since *cpn*60 is a protein-coding gene, all classical bioinformatic methods that evaluate DNA evolution in terms of point mutation frequencies are directly applicable to the analysis of *cpn*60 sequences, and sequence alignments are rapidly accomplished with established tools such as ClustalW [32]. Additionally, the lack of significant length variation in *cpn*60 UT sequences (185 amino acids +/−1 codon) makes it appropriate to use either global or local alignment methods when comparing sequences. By contrast, 16S rRNA genes encode structural RNA, necessitating the evaluation of mutations in the context of secondary structure using specialized algorithms such as INFERNAL [44]. Multiple sequence alignment tools such as the RDP Aligner and NAST [45] exploit methods to generate alignments based on comparison of input sequences to a reference alignment template, resulting in alignments that are not generally “human readable” due to large numbers of gaps. This sort of bioinformatic advantage is an important, but perhaps under-recognized aspect of a preferred barcode sequence.

The separation between distributions of intra- and inter-specific distances was originally termed a barcode gap [2], and while “the simplest test is whether genetic distances within species are less than those between species” [46], there is continuing debate about the best way to measure the barcode gap. Barcode gaps have been expressed in various ways including the difference between the smallest values in either distribution, the average distances, or the ratio of inter- and intra-specific distances [47]. Some authors have gone as far as recommending the establishment of defined cutoffs for barcode gaps [48]. Use of the difference between minimum distances, or ratio of inter- and intra-specific distances, was inappropriate in our case since the minimum distance in all of the intra- and inter-specific distributions was zero (Table 2). Instead we opted to compare the average intra- and inter-specific distances, using median values rather than mean since the distributions, especially the intra-specific distributions, were not normal. The use of the median value as the parameter of comparison between distributions has the additional advantage of reducing the influence of extreme values within the distributions due to factors discussed below.

While on first viewing, the presence of inter-specific zero distances may seem surprising, it is less so when one considers that the genome sequences examined included those from “problematic” taxa such as *Brucella* and *Bacillus*. It is known for these genera and others that historical definitions of species based on phenotypic properties are not always congruent with comparisons of phylogenetic markers such as 16S rRNA and cpn60 [49].

We included all annotated paralogs of 16S rRNA (median = 3 copies/genome) and cpn60 (median = 1 copy/genome) in our analysis since our interest in application of the barcoding concept in bacteria extends beyond the examination of isolates to characterization of complex microbial communities, where practitioners cannot select which paralogs of 16S rRNA or cpn60 are sequenced. In the case of 16S rRNA, paralogs are generally highly similar if not identical to one another [50], which tends to shift the intra-specific distance distribution toward zero (Figure 1). For *cpn*60, multiple copies per genome are the exception rather than the rule, and these paralogs are generally highly divergent, leading to the opposite effect of shifting the intra-specific distance distribution away from zero.

We were able to obtain sufficient data for thousands of intra-specific and millions of inter-specific comparisons from bacteria with complete genome sequences. The results of the barcode gap analysis (Figure 1, Table 2) revealed that among the longer 16S rRNA loci (those including 3 variable regions), V1–V3 had the largest gap. At 0.35, it is consistent with that of the ITS locus, recently proposed as a preferred barcode for fungi [3], and indicates that these loci do exhibit a barcode gap, albeit a small one compared to the other targets examined.

The difference between the two versions of the V6 target was striking, with the shorter (average 75 bp) version having a substantially larger barcode gap (0.59 *vs.* 0.26 for the 125 bp locus). This difference is likely accounted for by the fact that the shorter version of the locus is defined by PCR primers designed to exclude most of one of the adjacent conserved regions [30], which accounts for a substantial proportion of the 125 bp amplicon defined by Sundquist [5]. Short target regions such as the V6 regions of 16S rRNA have recently become more popular for gene-centric metagenomic studies that exploit short-read methods such as Illumina [30], [51], [52]. The disadvantage of these short targets is that they provide relatively few informative positions and may thus be substantially affected by PCR and sequencing error. Their short length also makes them limited in utility for the development of diagnostic methods for the detection of the corresponding organisms in complex samples.

Both the 75 bp V6 locus and the *cpn*60 UT had broad intra-specific distance distributions with long right-hand tails. In the case of the *cpn*60 UT, some of this is accounted for by the occurrence in some taxa of multiple *cpn*60 paralogs with highly divergent sequences. Although the median number of *cpn*60 copies per genome in our study was 1, and the norm in Bacteria is for a single copy per genome, the occurrence of multiple copies that are widely divergent in sequence is well known in some taxa including *Chlamydia,* some Rhizobia and some Actinobacteria [53], and these taxa were represented in the genome sequence collection examined in this study. Another contributing factor to the long right-hand tail in the intra-specific distance distribution for the *cpn*60 UT is the inclusion of some non- *cpn*60 but *cpn*60-related sequences as a result of the necessity of using multiple search terms to identify *cpn*60 gene annotations. Although there has been significant effort to standardize chaperonin nomenclature [37], [53], [54], current bacterial genome annotations often do not conform to these recommendations. An advantage to having *cpn*60 recognized as a barcode for Bacteria would be the standardization of annotations for this gene in bacterial genome sequences.

The *cpn*60 UT had the highest median intra-specific (0.07) and inter-specific (0.68) distances and the largest barcode gap of the loci examined (0.61, Table 2), clearly meeting the barcode evaluation criteria. However, an additional criterion is that the barcode be accessible with broad-range PCR primers. Although we exploited published sequence data rather than directly amplifying the target from bacterial isolates, there is a wealth of published studies of targeted analysis of particular taxa and un-targeted metagenomic studies to provide evidence of the efficacy of the broad-range (“universal”) PCR primers for the *cpn*60 UT. A review of the data within cpnDB that has been generated through application of the broad-range PCR primers shows that the distribution of the >150 distinct taxonomic lineages closely resembles the distribution in Table S1.

In addition to offering robust differentiation of bacterial species based on the examination of isolates, the *cpn*60 UT barcode can be exploited in high resolution profiling of microbial communities. Species level identifications are not often reported in 16S rRNA based metagenomic studies as a direct consequence of its frequent failure to differentiate bacterial species, and widely used tools such as the RDP classifier only provide identification to the genus level [55]. However, in some environments, species level resolution is desirable. For example, the human vaginal microbiome is dominated by *Lactobacillus* species and in some cases, special effort has been dedicated to resolving the common lactobacilli based on partial 16S rRNA sequences [30], [56]. In contrast, in *cpn*60 UT-based studies of the vaginal microbiome, species resolution was accomplished based on comparison of OTU sequences to a reference database using simple, rapid sequence comparisons [19].

In this study, we have demonstrated that the features of the *cpn*60 UT enable *de novo* assembly of OTUs, a process that has some important differences from more common clustering methods employed in gene-centric metagenomic sequence analysis. Current popular methods for OTU aggregation *via* clustering [57] form clusters of related sequences but do not yield a consensus sequence directly. Instead, clustering methods identify a representative sequence for each OTU by selecting either the nearest neighbour sequence in a reference database, the most common experimental sequence, or a sequence selected at random from the OTU constituents. These existing methods of OTU formation by clustering are useful, unsupervised methods to aggregate large amounts of experimental data but they do not empower discovery of novel OTUs.

The methods for OTU formation using sequence assembly we have described provide a framework for the assembly of full-length OTU consensus sequences in an unsupervised manner. We were able to reconstruct a synthetic community of 20 *cpn*60 UT sequences faithfully, and evaluate the quality of the results of different assembly strategies using an objective, quantitative measure (Figure 3). An examination of the results of assemblies of a single data set using a range of minimum overlap length and minimum identity values showed that there is a series of trade-offs involving the various types of error that may result from adjusting these settings. As shown in Figure 3, increasing the minimum overlap length can reduce the amount of total error in the final assembly, but there is a corresponding loss of raw data as the median read length is approached. Decreasing the minimum overlap length and/or the minimum identity could result in an increased likelihood of reads from closely related templates being inappropriately assembled into a single OTU. In the case of the 92% minimum identity assemblies, minimum overlap lengths of 100 and 150 result in low total error, and less than 5% data loss due to singletons, suggesting that this may represent a “sweet spot” for assembly parameters.

Optimal parameters could vary with different microbial population compositions, but the sensitivity and specificity metrics allow an objective assessment of the results of any assembly, even in the absence of knowledge of the actual composition of the community as we had with the synthetic community. Given that the OTU assembly procedure can be optimized to yield robust full-length barcode sequences with high specificity and sensitivity, it becomes possible to trust that if the assembly procedure yields novel sequences these can be relied upon as real biomarkers for an uncharacterized microbe (i.e. not represented among existing reference sequence data). Furthermore, the values for Sn and Sp for any individual OTU provide a potentially useful tool for the evaluation of the quality of a particular OTU of interest. Application of this concept has already resulted in the characterization of distinct subspecies groups within *Gardnerella vaginalis* that were originally identified based on assembly of metagenomic *cpn*60 UT data from human vaginal microbiota [19], [58].

The assemblies presented here are generated from 454 Titanium sequence data with an average read length of 394, which is typical of the 454 Titanium chemistry on the FLX and Junior platforms. It is anticipated the average read lengths for 454 pyrosequencing will consistently exceed 700 bp with the introduction of the FLX+ chemistry (Roche/454), which will most likely further improve *cpn*60 UT sequence assembly. We have not yet experimented with *cpn*60 UT sequencing on the Illumina platform, where average read length is commonly lower than that obtained with pyrosequencing. However current forecasts for read length suggest that Illumina’s MySeq platform may reach an average of 400 bp soon. The fact that sequence diversity is evenly distributed along the length of the *cpn*60 target suggests that even existing technologies that produce shorter reads would provide good discrimination of closely related taxa (Figure 2), even if full-length OTU assembly would not be possible. *De novo* assembly of 16S rRNA gene sequences would be significantly more difficult as the average sequence difference between species is in the range of technical errors, which may arise from PCR and sequencing protocols. The most informative regions of the 16S rRNA gene (corresponding to the V1–V3 regions where sequences have an average 96% identity to their closest match in the database) are less informative that the most conserved segments of the *cpn*60 UT, for which average sequence identity does not exceed 92% (Figure 2).

The results of our study indicate that the *cpn*60 UT provides a preferred barcode for Bacteria compared to the regions of the 16S rRNA gene we examined. The breadth of complete bacterial genome sequence data currently available is influenced by factors such as the cultivability of various taxa, and their relevance to human and animal health, and other well-explored environments. As this spectrum expands beyond what is currently available due to efforts to generate genome sequences for currently under-represented taxa [59], there will be continuing opportunities to evaluate barcoding potential of the *cpn*60 UT for these new taxa. However, based on the evidence to date, it is clear that the *cpn*60 UT barcode offers significant advantages for cataloging bacterial biodiversity through the analysis of isolates or in the context of microbial ecology studies. We suggest that *de novo* assembly of metagenomic sequence data from the *cpn*60 UT, or from any appropriate barcode sequence, is a useful approach, especially in cases where resolution beyond the genus level and the confident identification of potentially novel taxa is desirable. To support these activities, we are preparing to release a software package for metagenomic profiling using metagenomic assembly that provides a pipeline for the analysis of microbial profiling data using sequence assembly of barcodes, including the calculation of sensitivity and specificity.

Our results demonstrate that the Barcode of Life’s framework has relevance for a domain of life other than Eukaryota. Thus it is reasonable to consider the use of this framework for evaluating barcoding targets for Archaea, including 16S rRNA and the Type II chaperonin (ortholog of *cpn*60) [60].

## Supporting Information

Table S1 Taxonomic affiliations of the bacterial genomes used in the study.
(PDF)
