# Supplementary material for: The Chaperonin-60 Universal Target Is a Barcode for Bacteria That Enables De Novo Assembly of Metagenomic Sequence Data
Source: PLoS One. 2012 Nov 26;7(11):e49755. doi: 10.1371/journal.pone.0049755 (PMC3506640; doi:10.1371/journal.pone.0049755)
Supplement: Table S1 — Taxonomic affiliations of the bacterial genomes used in the study. (PDF) [file pone.0049755.s001.pdf]

Table S1. Taxonomic affiliations of the bacterial genomes used in the study.

Phylum*	Number of genomes (%)
Proteobacteria	661 (47.4)
Firmicutes	285 (20.4)
Actinobacteria	161 (11.5)
Bacteroidetes	69 (4.9)
Cyanobacteria	40 (2.9)
Spirochaetes	35 (2.5)
Chlamydiae	22 (1.6)
Chloroflexi	15 (1.1)
Thermotogae	13 (1.0)
Deinococcus-Thermus	13 (1.0)
Chlorobi	11 (0.8)
Tenericutes	10 (0.7)
Aquificae	10 (0.7)
Acidobacteria	7 (0.5)
Synergistetes	5 (0.4)
Planctomycetes	5 (0.4)
Fusobacteria	5 (0.4)
Verrucomicrobia	4 (0.3)
Deferribacteres	4 (0.3)
Thermodesulfobacteria	2 (0.1)
Nitrospirae	2 (0.1)
Dictyoglomi	2 (0.1)
Gemmatimonadetes	1 (0.1)
Fibrobacteres	1 (0.1)
Elusimicrobia	1 (0.1)
Chrysiogenetes	1 (0.1)
Unknown/Unassigned	9 (0.6)
TOTAL	1394 (100)

*Based on TaxID lookup in the NCBI Taxonomy database
